# Experimental Characterization of Viscoelastic Behaviors of Nano-TiO_2_/CaCO_3_ Modified Asphalt and Asphalt Mixture

**DOI:** 10.3390/nano11010106

**Published:** 2021-01-04

**Authors:** Chunli Wu, Liding Li, Wensheng Wang, Zhengwei Gu

**Affiliations:** College of Transportation, Jilin University, Changchun 130025, China; clwu@jlu.edu.cn (C.W.); lild17@mails.jlu.edu.cn (L.L.)

**Keywords:** nano-TiO_2_/CaCO_3_, bituminous materials, viscoelastic behaviors, dynamic shear rheometer, static compression creep, dynamic modulus

## Abstract

The purpose of this paper is to promote the application of nano-TiO_2_/CaCO_3_ in bituminous materials and present an experimental characterization of viscoelastic behaviors of bitumen and bituminous mixture modified by nano-TiO_2_/CaCO_3_. In this work, a series of viscoelastic behavior characterization tests were conducted, including dynamic shear rheometer (DSR) test for bitumen, uniaxial static compression creep test and dynamic modulus test for bituminous mixture. Moreover, various viscoelastic models with clear physical meanings were used to evaluate the influence of nano-TiO_2_/CaCO_3_ on the macroscopic performance of bitumen and bituminous mixture. The results show that bitumen and its mixtures are time-temperature dependent. The Christensen-Anderson-Marasteanu (CAM) model of frequency sweep based on DSR test indicated that adding nano-TiO_2_/CaCO_3_ can effectively capture the sensitivity of temperature. In addition, the incorporation of nano-TiO_2_/CaCO_3_ in bituminous mixture can significantly enhance the high-temperature anti-rutting, and slightly improve the low-temperature anti-cracking as well. At the same time, the modified Burgers model can accurately describe the viscoelastic behavior of bituminous mixtures in the first two creep stages, reflecting the consolidation effect of bituminous mixture. Also, the generalized Sigmoidal model can accurately grasp the characteristics of the relationship between dynamic modulus and reduced frequency and achieve good prediction effects in a wider frequency range.

## 1. Introduction

Due to superior performance, bituminous pavement has become the most common pavement in China’s high-level pavements [1,2,3]. With the development of transportation, bituminous pavement related technology and measurements have been continuously developed, and its service performance and level have been significantly improved [4,5,6,7,8]. However, it is worth noting that there are yet many problems in the field of flexible pavement that need to be solved urgently. Bituminous material has a significant feature that its properties are strongly influenced by its service temperature [9,10,11]. Damage resulting from extremes in temperature will reduce the service performance of bituminous flexible pavement, as evident in rutting, cracks and other defects [2,12,13].

There are many factors affecting the performance degradation of bituminous flexible pavement, including material internal factors and service condition factors [14]. For the sake of improving the mechanical properties of bituminous flexible pavement, a lot of related research work has been done, including modification of bituminous materials [15,16], optimizing bituminous flexible pavement structure [17]. However, with the rapid development of nanotechnology, more and more researchers are committed to introducing nanomaterials to modify bitumen [18]. Nanomaterials refer to materials in the range of 1~100 nanometers in at least one dimension. It has previously been observed that the physical, chemical and other properties of nanomaterials have great differences with the original raw materials [19]. It is worth noting that nanomaterials usually have the advantages of significant temperature susceptibility, better extendability and larger specific surface area (SSA). Therefore, on the above basis, researchers introduced nanomaterials into road and construction fields. Jahromi et al. employed two kinds of nano-clay to improve the performance of bituminous materials. According to X-ray diffraction, along with dynamic shear rheometer (DSR) tests, it was found that the nano-clay modified bitumen increased stiffness and decreased phase angle [20]. Abdelrahma et al. assessed the physical performances of bitumen through adding the modified nano-clay using dynamic mechanical analysis and showed that the incorporation of modified nano-clay materials into bituminous materials enhanced their physical properties. Also, they investigated the modification mechanism of nano-clay, which was considered to be the interactivity of the modified nano-silox tetrahedron in bitumen using FTIR test [21]. You et al. used nano-clay to modify bitumen and compared two kinds of nano-clay. The results indicated that nano-clay could effectively boost the comprehensive performance of bituminous materials. Furthermore, the blending procedure was considered as the key to achieving a well-distributed nano-clay modified bitumen [22]. Khattak et al. employed different dosages of carbon nanofibers to modified three types of bituminous cements based on two bituminous mixing procedures, i.e., dry and wet procedures. Due to the larger SSA, better interface combination effect, as well as higher modulus values of carbon nanofiber, the test results showed that carbon nanofiber modified bitumen exhibited good viscoelastic response and fatigue performances [23]. Filho PG et al. applied different contents of nano-TiO_2_ to base asphalt binder with penetration grade 50/70. Through multiple stress creep recovery, linear amplitude sweep and conventional tests, they found that nano-TiO_2_ could improve the fatigue resistance [24]. Besides, they also concluded that nano-TiO_2_ addition demonstrated a delay on ageing of asphalt [25]. Chen et al. utilized nano-TiO_2_ to modify bitumen through permeability technology, and evaluated the penetration effect using scanning electron microscope. Due to the large surface area and advanced oxidation technology of nano-TiO_2_, nano-TiO_2_ modify bitumen produced good performances of bitumen and also had good environment purification function [26]. Due to the large SSA, good dispersion as well as stability of nano-silica, it was widely used in the fields of medicine, engineering and so on. It was found that the performances of bituminous materials were greatly enhanced through incorporating nano-silica [27]. Yusoff et al. found that the susceptibility to moisture damage of polymer modified bituminous materials was decreased while their anti-rutting and fatigue performance were increased through incorporating nano-silica [28]. Using the mentioned nano materials could significantly boost the ability of bituminous flexible pavement to meet the requirements of service conditions; for instance, anti-rutting and anti-cracking [29,30].

Despite all that, considering the typical visco-elastic-plastic characteristics of bituminous flexible pavement under its service conditions, there are still inescapable deformations [31,32,33]. Despite various technical measures, there are still many problems related to deformation resistance of bituminous flexible pavement, including ruts, cracks and other deformation damage phenomena, which can be attributed to the insufficient deformation resistance [34,35]. Consequently, it is quite essential to discuss and evaluate the deformation performance of bituminous materials from the perspective of a viscoelastic constitutive model. Liu et al. proposed two methods based on the Kramers-Kronig relations. They constructed the master curve models with four viscoelastic parameters for bituminous mixtures by these two methods [36]. Lagos-Varas et al. developed a new method of viscoelastic mechanical behaviors based on derivatives of fractional order. This method can well describe the practical construction and be suitable for modified bitumen [37]. Wang et al. prepared the polymer and basalt fiber modified bituminous mixtures by Superpave gyratory compaction. Then they evaluated the influences of freeze-thaw cycles on the viscoelastic properties [38]. In order to investigate the influences of various ingredients on the viscoelastic behavior of bituminous materials, Ma et al. performed laboratory tests and virtual creep test based on discrete element method [39]. Darabi et al. investigated the nonlinear viscoelastic, viscoplastic and hardening-relaxation of bituminous mixture using a proposed systematic analysis means. Then they applied dynamic modulus and repeated creep recovery tests to verify the mechanical response of the proposed method [40].

The purpose of the current work was to evaluate the application of nano-TiO_2_/CaCO_3_ in bituminous materials and presented an experimental characterization of viscoelastic behaviors of bitumen and bituminous mixture modified by nano-TiO_2_/CaCO_3_. Compared to the control base bituminous materials, a series of viscoelastic behavior characterization experiments were carried out, including dynamic shear rheometer (DSR) test for bitumen, uniaxial static compression creep test and dynamic modulus test for bituminous mixture. Moreover, various viscoelastic models with clear physical meanings were used to discuss the influences of nano-TiO_2_/CaCO_3_ on the macroscopic performance of bituminous materials.

## 2. Raw Materials and Experimental Methods

### 2.1. Raw Materials and Tested Specimens

#### 2.1.1. Raw Materials

1. Base Bitumen

The 90# base bitumen (AH-90, with the penetration at 25 °C of 80–100/0.1 mm) was acquired from the Panjin Petroteum Asphalt Co., Ltd. (Panjin, Liaoning Province, China). Its main technical properties are shown in Table 1.

2. Nano-TiO_2_/CaCO_3_

The nano-TiO_2_/CaCO_3_ was developed and provided by the college of chemistry, Jilin University [41]. Its detailed technical characteristics are presented in Table 2.

3. Aggregates and Mineral Filler

The coarse and fine aggregates were acquired by crushing basalt stone from Jiutai City, Jilin Province for later preparation of bituminous mixtures. In addition, the filler used in the bituminous mixture was limestone powder from Antu City, Jilin Province. Table 3 shows the main technical properties of coarse and fine aggregates and limestone powder in this paper, which meets the requirements of the specification JTG F40-2004.

#### 2.1.2. Preparation of Nano-TiO_2_/CaCO_3_ Modified Bitumen and Bituminous Mixture

Prior studies that have noted the reasonable dosage of nano-TiO_2_/CaCO_3_ is 5% in weight of bitumen [41]. During the preparatory stage of nano-TiO_2_/CaCO_3_ modified bitumen, original bituminous material was preheated to 160 °C and next it was blended with modified nano-TiO_2_/CaCO_3_ by manually stirring for 5 min. The corresponding temperature increased to 170 °C in a short time. Finally, the high-speed shearing was carried out with a speed of 6000 r/min at 170 °C for 40 min. Before use, heat the bituminous sample again to 170 °C, and control the shearing speed at 450~600 r/min, and stir continuously for 20 min.

**Figure 1:**
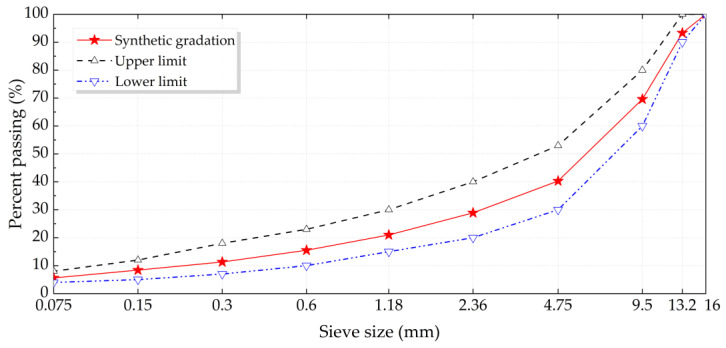
Bituminous mixture gradation curve in this paper.

In addition, the coarse aggregate voids-filling method (CAVF) was adopted to design the gradation of bituminous mixtures, including base bitumen and nano-TiO_2_/CaCO_3_ modified bitumen, and the gradation curve is illustrated in Figure 1 [42]. According to the specification JTG E20-2011, the optimum asphalt-aggregate ratios of base original bituminous concrete as well as nano-TiO_2_/CaCO_3_ modified bituminous concrete were obtained by Marshall design method. Marshall stability, flow, air voids, etc. have been comprehensively considered for different asphalt-aggregate composition from 4.0% to 6.0% with an interval of 0.5% [17,43]. The asphalt-aggregate ratio of base original bituminous concrete as well as nano-TiO_2_/CaCO_3_ modified bituminous concrete were determined as 4.9% and 5.3% by the weight of the aggregates, respectively.

### 2.2. Laboratory Tests

#### 2.2.1. Dynamic Shear Rheometer Test of Bitumen

**Figure 2:**
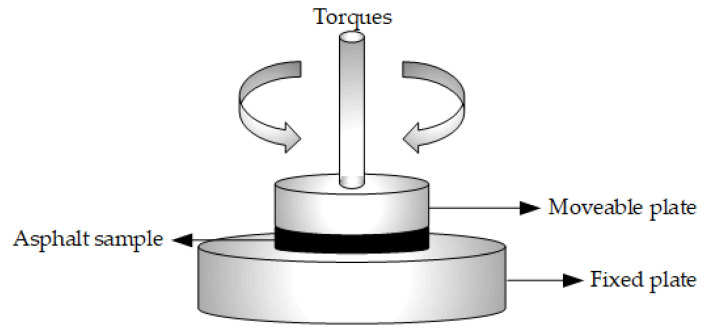
DSR loading method of bituminous sample.

The DSR test developed by SHRP is employed to analyze the dynamic characteristics and evaluate the viscoelastic behavior of asphalt materials [44,45,46]. Compared to static experiments (penetration, softening point, etc.), the DSR test has more intuitive and real advantages to assess the properties of bituminous materials. According to the specification ASTM D7175 (AASHTO T31509), the rheological parameters of bituminous materials are determined by Malvern Bohlin Gemini 150 (British Malvern Instruments Ltd.). As shown in Figure 2, by using two parallel plates at the temperature, the DSR test is carried out under constant strain mode at 10 rad/s.

In the DSR test, the dynamic viscoelastic characteristics of bitumen can be divided into two parts, i.e., complex shear modulus (G*) and phase angle (*δ*). The complex shear modulus (G*) is generally calculated by applying dynamic shear stress (*τ*_max_) to bituminous sample and the corresponding measured shear strain (*γ*_max_), defined in the Equation (1). The phase angle (*δ*) reflects the ratio of viscoelasticity in bitumen. When at higher temperatures or lower-frequency loading, bitumen is more prone to viscous flow, so the phase angle is larger. While at lower temperature or higher-frequency loading, bitumen exhibits more elastic properties and the phase angle is smaller.

(1) $$G^{*}=\frac{\tau_{\max}}{\gamma_{\max}}$$

#### 2.2.2. Uniaxial Static Compression Creep Test

The creep test methods mainly include uniaxial static compression creep, bending creep and splitting creep, dynamic triaxial compression creep. At present, the commonly used creep test methods in the world for bituminous mixture are mainly uniaxial static compression creep and bending creep, in which the uniaxial static compression creep test is the simplest and most practical method. A major advantage of uniaxial static compression creep test is that the test equipment is relatively simple, therefore, this creep test method has been widely used [38,47].

**Figure 3:**
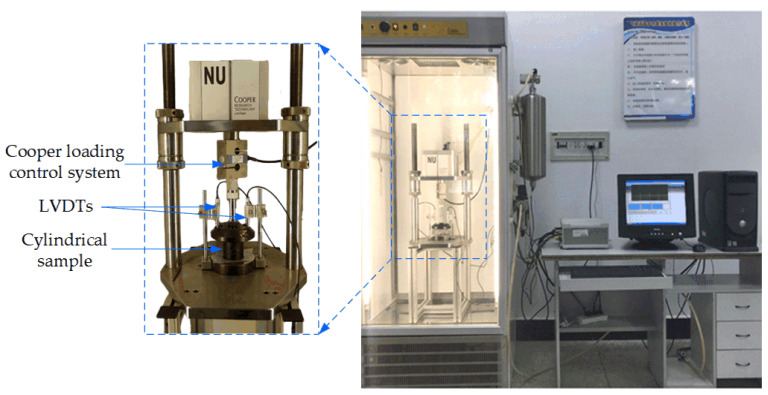
Uniaxial static compression creep test by Cooper NU-14 used in this paper.

In this paper, the uniaxial static compression creep experiment was performed for base original bituminous concrete and nano-TiO_2_/CaCO_3_ modified bituminous concrete specimens using a NU-14 tester, whose sensor measurement accuracy is 0.001%. Before the test, a smooth polytetrafluoroethylene (PTFE) plastic film was placed on the upper and lower surfaces of bituminous mixture sample to eliminate or reduce the influence of friction on contact surfaces. Meanwhile, bituminous mixture samples should be kept in an environmental chamber for more than 4 h to ensure a uniform sample temperature. Figure 3 exhibits the uniaxial static compression creep test, and both sides of test samples are required to be flat to prevent local stress concentration from affecting the deformation response. At the beginning of the creep test, a loading of 0.002 MPa was preloaded first, and then the loading with a stress level of 0.3 MPa for 2700 s as well as unloading for 1800 s was carried out. During the creep test, the deformation data of samples were collected by LVDT sensors.

#### 2.2.3. Dynamic Modulus Test

Dynamic mechanical analysis has been used in the past to investigate the viscoelastic properties of bituminous concretes. Dynamic modulus test is one of the most common procedures for determining the dynamic modulus of bituminous mixture [48,49]. In this paper, the dynamic modulus experiment was carried out for three replicate specimens at different experimental conditions, the detailed test program is shown in Table 4 and Table 5.

**Figure 4:**
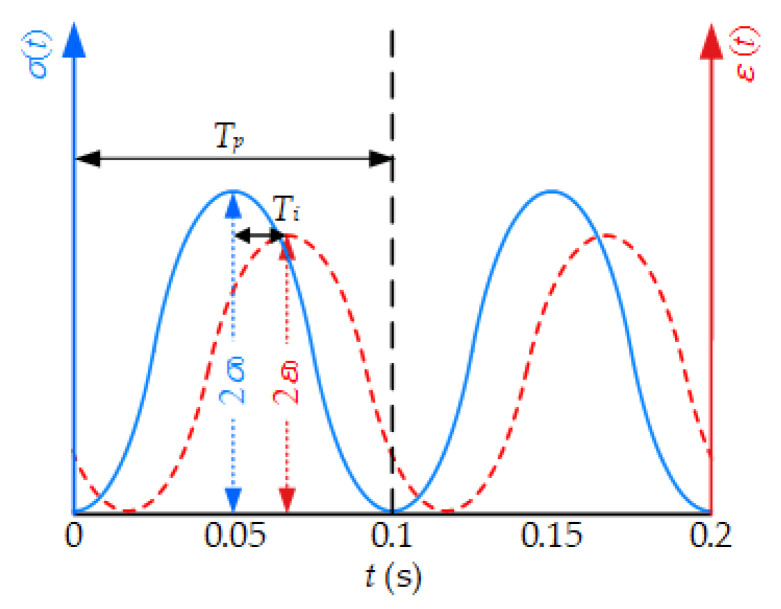
The mechanical response in dynamic modulus test.

Based on the collected stress and strain data, the complex modulus (E*) can be calculated, which characterizes their relationship subjected to semi-sine load. The complex modulus is related to the corresponding maximum values (2*σ*_0_ and 2*ε*_0_) of sine wave at a given time (*t*) and angular frequency (*ω*), as expressed in the Equation (2). The mechanical response is shown in Figure 4 [50].

(2) $$E^{*}=\frac{\sigma_{0}\sin(\omega t)}{\varepsilon_{0}\sin(\omega t-\delta )}$$

The dynamic modulus $\left|E^{*}\right|$ is the absolute value of E*, and the characteristics (*δ*) describes the relative lag of the viscous and elastic parts of bituminous material, written as the Equations (3) and (4).

(3) $$\left|E^{*}\right|=\frac{\sigma_{0}}{\varepsilon_{0}}$$

(4) $$\delta =\frac{t_{i}}{t_{p}}$$

where *t_i_* is the average retardation time in the last five cycles, *t_p_* is the average load period of the last five loading cycles.

## 3. Results and Discussion

### 3.1. Dynamic Shear Rheometer Test of Bitumen

#### 3.1.1. Complex Shear Modulus (G*)

**Figure 5:**
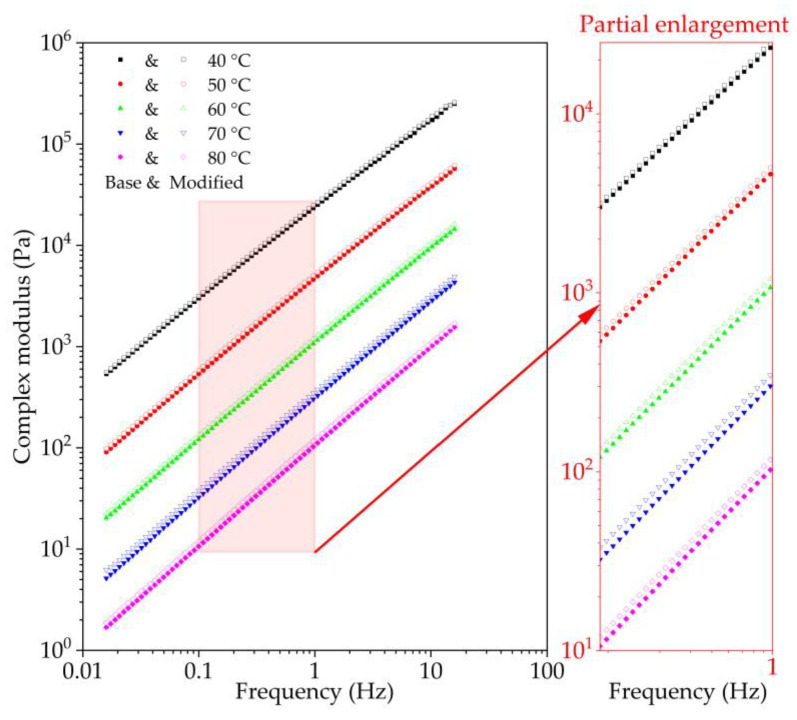
Complex modulus results versus frequencies and temperatures for base bitumen and nano-TiO_2_/CaCO_3_ modified bitumen.

The frequency sweep test is currently the most popular method for investigating viscoelastic mechanical parameters of bitumen. The dynamic shear modulus mechanical response of bitumen in the linear viscoelastic range can be obtained using DSR test with small strain level under different loading frequencies at the test temperature. To explore the viscoelastic properties of nano-TiO_2_/CaCO_3_ modified bitumen at higher temperature, the frequency sweep experiment was conducted based on DSR test from 40~80 °C with an interval temperature of 10 °C for three replicate samples of base bitumen and nano-TiO_2_/CaCO_3_ modified bitumen. Before the frequency sweep test, bitumen needs to be kept at the test temperature for at least 15 min. The measured complex modulus (G*) varying with frequency are shown in Figure 5.

As seen in Figure 5, as loading frequency increases, the complex shear modulus of both base bitumen and nano-TiO_2_/CaCO_3_ modified bitumen increases, and shows a linear growth trend in the logarithmic coordinate. Simultaneously, nano-TiO_2_/CaCO_3_ modified bitumen has a slightly higher complex shear modulus than base bitumen at the same frequency.

#### 3.1.2. Master Curve Analysis of Complex Shear Modulus

In DSR, the loading frequency is generally selected as 0.1~100 rad/s. Then, based on the principle of time-temperature equivalence, the complex shear modulus data at different test temperatures can be shifted horizontally, and a master curve of complex shear modulus is obtained to characterize its linear viscoelastic properties [45].

The Christensen-Anderson-Marasteanu (CAM) model is adopted as fitting equation of master curve of complex shear modulus, shown as below:

(5) $$\left|G^{*}\right|=\frac{\left|G_{g}^{*}\right|}{{\left[1+{\left(f_{c}/f^{\prime }\right)}^{k}\right]}^{m/k}}$$

where $\left|G_{g}^{*}\right|$ is the glassy shear modulus of bitumen and set as 10^9^ Pa in this paper, *k* and *m* represent fitting terms, *f*’ and *f_c_* are the reduced frequency and actual loading frequency, respectively.

Based on the traditional Williams-Landel-Ferry (WLF) equation, the shift factor (*α_T_*) can be obtained as shown in the Equation (6) [48,50].

(6) $$\log\alpha_{T}=-\frac{p_{1}(T-T_{0})}{p_{2}+(T-T_{0})}$$

in which *p*_1_ and *p*_2_ represent fitted terms, *T*_0_ is the reference temperature.

**Figure 6:**
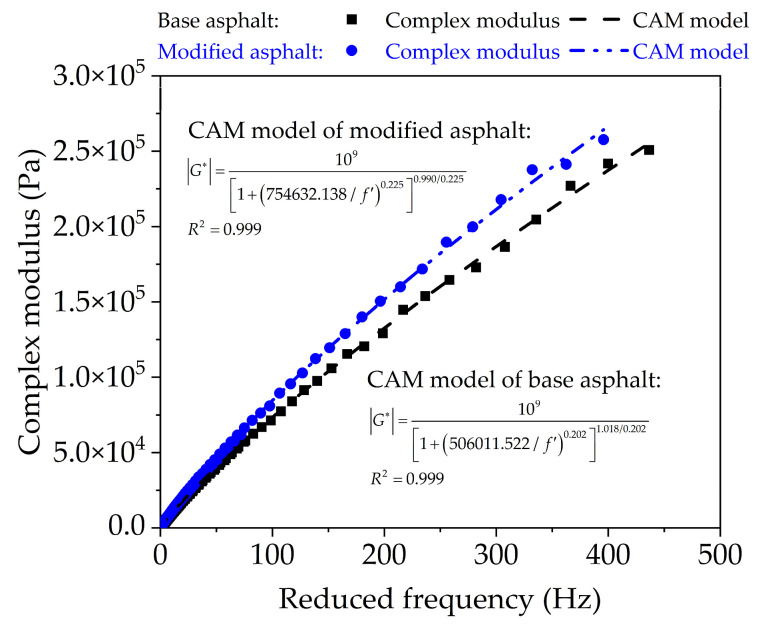
The master curve of dynamic modulus for base bitumen and nano-TiO_2_/CaCO_3_ modified bitumen based on CAM model.

Taking 60 °C as the reference temperature, the shift factors (*α_T_*) of base bitumen and nano-TiO_2_/CaCO_3_ modified bitumen at different temperatures are shown in Table 6. Based on the CAM model equation, the complex shear modulus and CAM model are plotted in Figure 6.

As presented in Figure 6, complex modulus of base bitumen and nano-TiO_2_/CaCO_3_ modified bitumen are frequency dependent, and their complex modulus increases with reduced frequency. At the same reduced frequency, the complex modulus of nano-TiO_2_/CaCO_3_ modified bitumen is higher. Moreover, the higher the frequency, the more significant their difference. Since the frequency relates to temperature, it also indicates that nano-TiO_2_/CaCO_3_ could boost the stabilization capability at higher temperature. In addition, CAM model is able to better fit the complex modulus of base original bitumen and nano-TiO_2_/CaCO_3_ modified bitumen with frequency. The fitting parameter *m* generally represents the sensitivity of bitumen to frequency, and the smaller the value of *m*, the lower the sensitivity of bitumen to frequency. Thus, the addition of nano-TiO_2_/CaCO_3_ reduced the temperature sensitivity of bitumen.

### 3.2. Uniaxial Static Compression Creep Test

#### 3.2.1. Uniaxial Static Compression Creep Test

(1) Creep deformation

**Figure 7:**
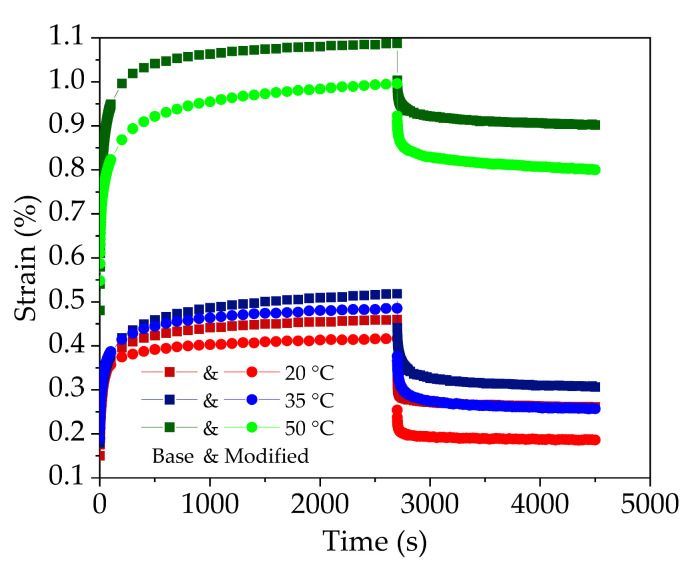
Creep deformations versus time for base bituminous mixture and nano-TiO_2_/CaCO_3_ modified bituminous mixture at 20 °C, 35 °C and 50 °C.

Taking into account the climatic characteristics of the seasonal freezing zone in Northeast China, the uniaxial static compression creep tests at 20 °C, 35 °C and 50 °C were carried out on three replicate specimens of base bitumen and nano-TiO_2_/CaCO_3_ modified bitumen. The creep deformation results versus time are plotted in Figure 7.

From Figure 7, it shows that base bituminous mixture and nano-TiO_2_/CaCO_3_ modified bituminous mixture have similar creep deformation curves. At the loading stage, the creep deformation includes instant and delayed elastic as well as viscous flow deformations, while at unloading stage, creep deformation includes instant and delayed elastic recovery deformation as well as permanent deformation. Although incorporating nano-TiO_2_/CaCO_3_ will not change the creep deformation law of bituminous mixture, nano-TiO_2_/CaCO_3_ could affect the creep deformation rate, cumulative deformation and residual permanent deformation.

**Figure 8:**
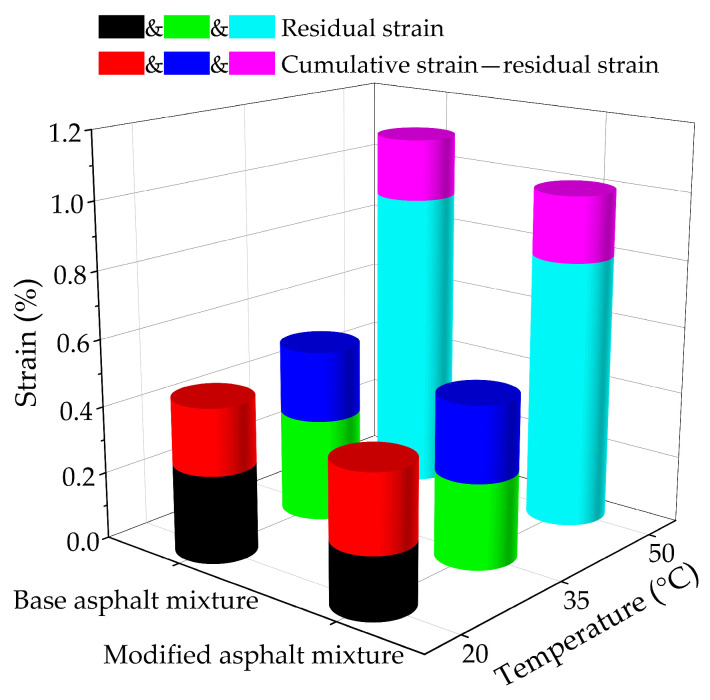
The residual strain ratio of base bituminous mixture and nano-TiO_2_/CaCO_3_ modified bituminous mixture at different test temperatures.

Figure 8 summarizes the cumulative strain and residual strain during the creep test for base original bituminous concrete and nano-TiO_2_/CaCO_3_ modified bituminous concrete at various test temperatures. The cumulative, as well as residual strain values of nano-TiO_2_/CaCO_3_ modified bituminous concrete are smaller at the same temperature, which represents nano-TiO_2_/CaCO_3_ can boost the deformation resistance of bituminous concrete at higher temperature.

(2) Creep Stiffness Modulus

Bituminous concrete is a typical type of viscoelastic material, and its stiffness modulus is a function of time (*t*) and temperature (*T*). In the uniaxial static compression creep test, the applied stress is a constant value (*σ*), its creep stiffness modulus (*S_m_*) versus strain *ε*(*t*,*T*) can be expressed in the Equation (7)

(7) $$S_{m}(t,T)=\sigma /\varepsilon (t,T)$$

**Figure 9:**
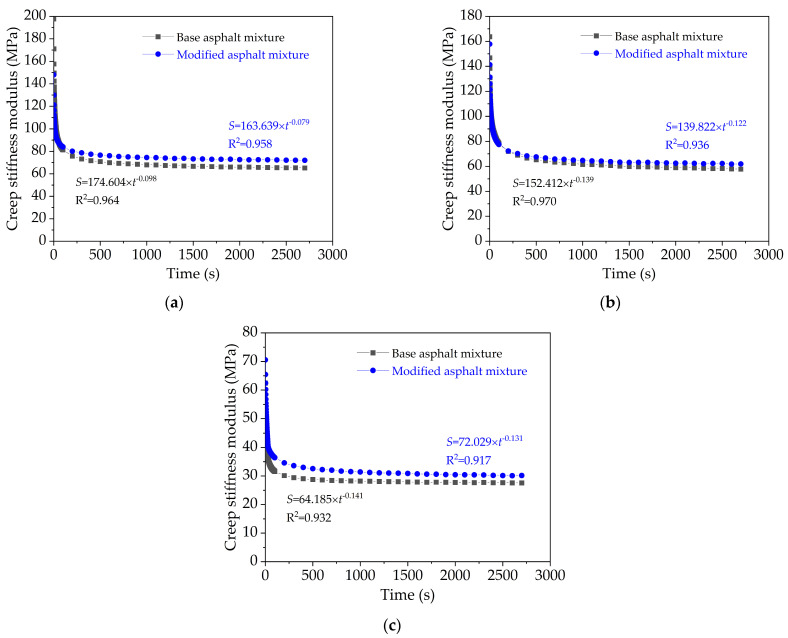
Creep stiffness modulus versus time for base bituminous mixture and nano-TiO_2_/CaCO_3_ modified bituminous mixture: (**a**) 20 °C; (**b**) 35 °C; and (**c**) 50 °C.

Figure 9 plots the creep stiffness modulus curves versus time for base original bituminous concrete and nano-TiO_2_/CaCO_3_ modified bituminous concrete at various test conditions. Evidently, creep stiffness modulus of both two bituminous mixtures decrease with loading time, but nano-TiO_2_/CaCO_3_ modified bituminous concrete has a higher creep stiffness modulus than base original bituminous concrete, indicating that incorporating nano-TiO_2_/CaCO_3_ would enhance the high temperature property of bituminous concrete.

In addition, through power function fitting analysis, it is clear that nano-TiO_2_/CaCO_3_ modified bituminous mixture has a smaller absolute value of power parameter in the fitting equations, which represents the incorporation of nano-TiO_2_/CaCO_3_ reduces the variation rate of creep stiffness modulus of bituminous mixture.

(3) Creep Activation Energy

Creep curve describes the deformation properties of bituminous concrete while testing. Prior studies have shown that the creep rate tends to be stable at the second stage of creep, which is called creep stable stage. The second stage lasts for a long time and has the greatest effect on permanent deformation of bituminous concrete. Creep curve slope (*k*) is the steady-state creep rate of bituminous mixture at the second stage, which is related to the material characteristics and test temperature. Generally, the larger the slope (*k*), the faster the deformation produced under loading and the worse the resistance to deformation. Moreover, The relationship between slope (*k*) and absolute temperature (*T*) can be expressed as the Arrhenius form:

(8) $$k=A_{2}\exp(-Q_{c}/RT)$$

where *Q_c_* represents creep activation energy, *R* is 8.314 J/(mol·K), *A*_2_ is material constant.

The creep curve slope (*k*) and creep activation energy are presented in Table 7. As the temperature increases, the steady-state creep rate (*k*) of both two bituminous mixtures increase. However, the slope (*k*) value of nano-TiO_2_/CaCO_3_ modified bituminous concrete is lower. Meanwhile, incorporating nano-TiO_2_/CaCO_3_ could improve the creep activation energy of bituminous mixture significantly. In other words, the deformance resistance of bituminous concrete at higher temperature has been greatly improved.

#### 3.2.2. Creep Model Analysis of Bituminous Mixture

The viscous and elastic elements are generally combined in series or in parallel to represent the viscoelastic mechanical performances of bituminous concretes, and Burgers model as well as its modified model are widely used and have good application effects [38,47]. The creep functions of both models are given as follow:

(9) $$\mathrm{Burgers}\;\mathrm{model}:\;\varepsilon (t)=\sigma_{0}\left[\frac{1}{E_{1}}+\frac{t}{\eta_{1}}+\frac{1}{E_{2}}\left(1-e^{-E_{2}t/\eta_{2}}\right)\right]$$

(10) $$\mathrm{Modified}\;\mathrm{Burgers}\;\mathrm{model}:\;\varepsilon (t)=\sigma_{0}\left[\frac{1}{E_{1}}+\frac{\left(1-e^{-Bt}\right)}{AB}+\frac{1}{E_{2}}\left(1-e^{-E_{2}t/\eta_{2}}\right)\right],$$

where *E*_1_, *E*_2_, *η*_1_, and *η*_2_ are viscoelastic parameters, *A* and *B* are fitting constants.

**Figure 10:**
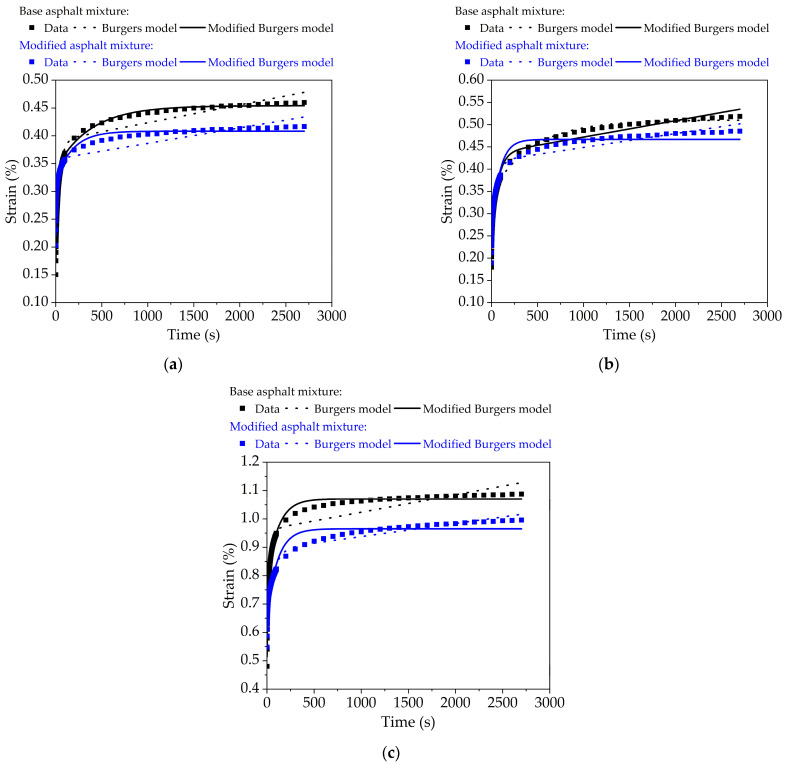
Comparative results of Burgers model and modified Burgers model for bituminous mixtures versus time: (**a**) 20 °C; (**b**) 35 °C; and (**c**) 50 °C.

Figure 10 plots the fitting curves of creep deformation for base original bituminous concrete and nano-TiO_2_/CaCO_3_ modified bituminous concrete at different test temperatures based on both models. As seen from Figure 10, the modified Burgers model are closer to actual measured creep deformation data, and the fitting accuracy is higher. The modified Burgers model could consider the consolidation effect of bituminous concrete, that is, the creep growth rate of bituminous mixture gradually decreases in the actual creep process. However, the Burgers model has good fitting results at the early stage of creep, but the creep deformation is gradually different from the actual deformation after the creep migration period. Therefore, the Burgers model is more ideal and the modified Burgers model is closer to reality.

### 3.3. Dynamic Modulus Test

#### 3.3.1. Uniaxial Compression Dynamic Modulus Test

(1) Dynamic Modulus

In this paper, dynamic modulus experiment was carried out on mechanical testing & simulation (MTS) test system at test conditions listed in Table 5. And this test was carried out in the order of increasing temperature and decreasing frequency.

**Figure 11:**
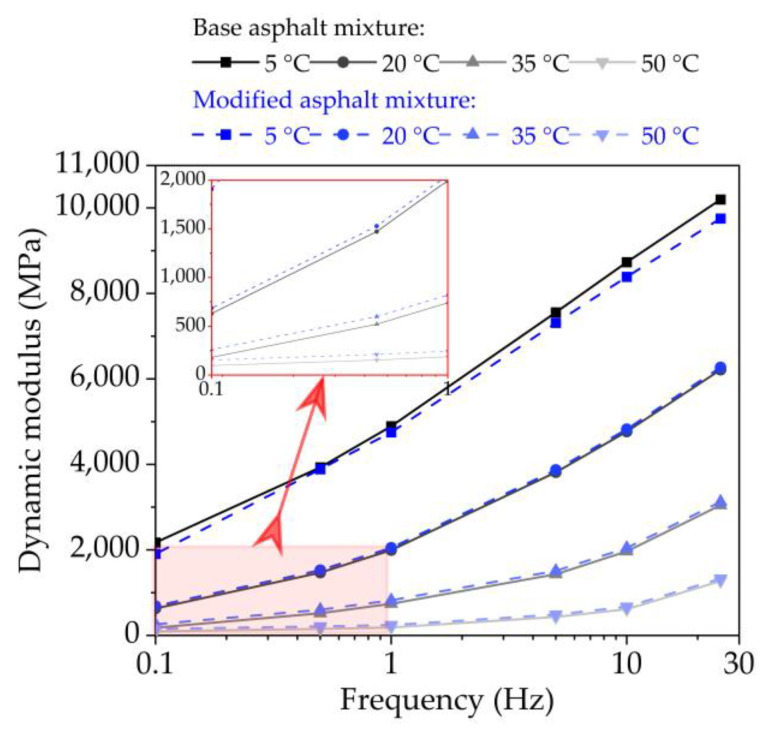
The dynamic modulus of base bituminous mixture and nano-TiO_2_/CaCO_3_ modified bituminous mixture at different test temperatures.

Figure 11 presents the dynamic modulus of base original bituminous concrete as well as nano-TiO_2_/CaCO_3_ modified bituminous concrete at various test conditions. It is observed intuitively in Figure 11 that the dynamic modulus of both bituminous concretes increase significantly with frequency, but the growth rate of dynamic modulus slows down gradually, which shows that the dynamic modulus of bituminous concretes will not increase indefinitely with frequency. The base bituminous mixture has higher dynamic modulus than nano-TiO_2_/CaCO_3_ modified bituminous mixture at lower temperature, while the opposite result at higher temperatures.

(2) Phase Angle

**Figure 12:**
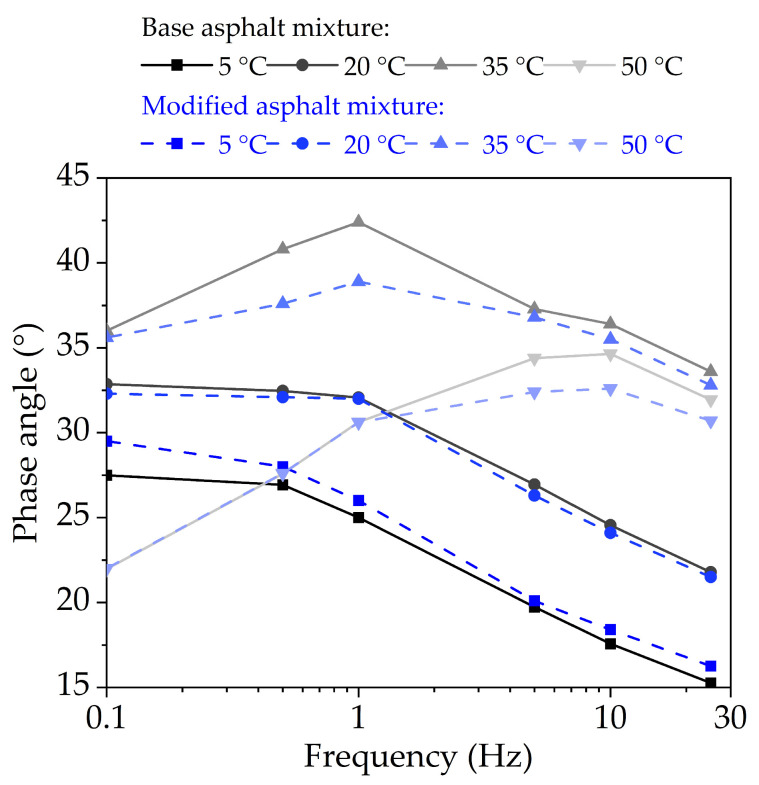
The phase angle of base bituminous mixture and nano-TiO_2_/CaCO_3_ modified bituminous mixture at different test temperatures.

Generally, the viscoelastic ratio of bituminous mixture increases with the increasing of test temperature or decreasing of loading frequency, which means that the phase angle should increase. Figure 12 shows the phase angle of base original bituminous concrete and nano-TiO_2_/CaCO_3_ modified bituminous concrete at different test temperatures. It can be seen that when the test temperature ≤20 °C, the phase angle (*δ*) changes of both two bituminous mixtures and conforms to this law. At 35 °C and above, the values of *δ* first increase and then decrease. This is because bituminous concrete is more influenced by the bituminous binder at lower temperatures or high-frequency loading. While at high temperatures or low-frequency loading, the mineral skeleton plays an important role for bituminous mixture. Due to the phase angle 0° of elastic aggregates, the phase angle of bituminous mixture will drop.

#### 3.3.2. Master Curve Analysis of Dynamic Modulus

Viscoelastic materials are dependent on time and temperature, that is, increasing temperature and extending time have equivalent effect with decreasing temperature and shortening time for the viscoelastic characteristics, i.e., the principle of time-temperature equivalence. Based on this, the measured dynamic modulus of bituminous mixture in dynamic modulus test at various test conditions can be converted into the values at the reference temperature using the shift factor, thereby forming a smooth master curve of dynamic modulus. Therefore, the viscoelastic behavior of bituminous concretes in a larger temperature and frequency interval would be forecasted according to the master curve of dynamic modulus [48,50].

As mentioned in the literature review, the generalized Sigmoidal model can well characterize the master curve of bituminous mixture, as shown below:

(11) $$\mathrm{lg}\left|E^{*}\left(f_{r}\right)\right|=\delta +\frac{\alpha -\delta }{{\left(1+\lambda \cdot e^{\beta +\gamma \mathrm{lg}f_{r}}\right)}^{\frac{1}{\lambda }}}$$

**Figure 13:**
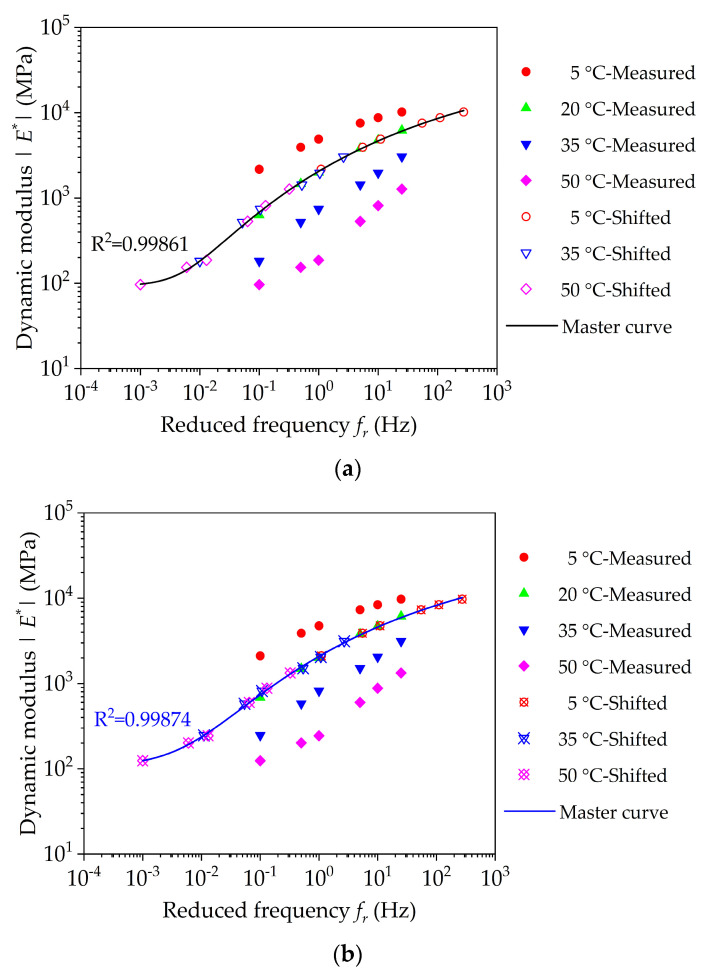

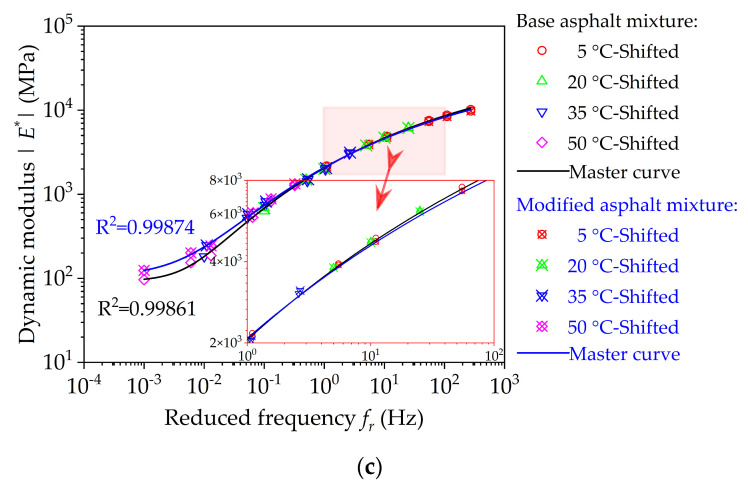
Master curve results of dynamic modulus (reference temperature = 20 °C): (**a**) base bituminous mixture; (**b**) nano-TiO_2_/CaCO_3_ modified bituminous mixture; and (**c**) comparative results.

Figure 13 presents the shifted dynamic modulus along with measured dynamic modulus at 20 °C. From Figure 13, the dynamic modulus of nano-TiO_2_/CaCO_3_ modified bituminous concrete is larger at low frequencies, and the corresponding value is 8~25% larger compared to base bituminous concrete at 50 °C. This shows that incorporating nano-TiO_2_/CaCO_3_ greatly improves the high-temperature rutting resistance of bituminous concrete, which is consist with the analysis results of creep stiffness modulus. While at high frequencies, the dynamic modulus of nano-TiO_2_/CaCO_3_ modified bituminous concrete is lower, and compared to base bituminous concrete, the corresponding values are also lower by about 1~4% at 5 °C. Therefore, it can be considered that the incorporation of nano-TiO_2_/CaCO_3_ could boost the anti-cracking of bituminous concrete at lower temperature to some extent.

According to the generalized Sigmoidal model in Equation (11), the master curves of dynamic modulus of base bituminous concrete and nano-TiO_2_/CaCO_3_ modified bituminous concrete at 20 °C are also plotted in Figure 13. It can be seen that the fitting generalized Sigmoidal models could correctly grasp the relationship characteristics of the two, which has higher correlation coefficient R^2^ close to 1. Generally, the lower frequency range and higher frequency range can indirectly reflect the high temperature and low temperature performances. From the comparison of master curves between base bituminous concrete and nano-TiO_2_/CaCO_3_ modified bituminous concrete, it is also observed that incorporating nano-TiO_2_/CaCO_3_ could significantly enhance the high-temperature rutting resistance of bituminous concrete, and the change in low-temperature crack resistance was not apparent. In addition, within the entire frequency range, the dynamic modulus of nano-TiO_2_/CaCO_3_ modified bituminous mixture has a relative smaller variation, indicating that nano-TiO_2_/CaCO_3_ modified bituminous mixture is less sensitive to temperature.

## 4. Conclusions

In this work, composite nanomaterials (nano-TiO_2_/CaCO_3_) were used to modify base bitumen, and then the nano-TiO_2_/CaCO_3_ modified bitumen was used to prepare bituminous mixture. In addition, the rheological properties, and dynamic and static viscoelastic characterizations of base bituminous mixture and nano-TiO_2_/CaCO_3_ modified bituminous mixture were tested and analyzed. The following conclusions are drawn:

(1) The frequency sweep based on DSR test indicated that the complex modulus of both base bitumen and nano-TiO_2_/CaCO_3_ modified bitumen are frequency dependent. Moreover, the addition of nano-TiO_2_/CaCO_3_ can effectively reduce the temperature sensitivity of bitumen, as reflected in the master curve of complex shear modulus base on CAM model.
(2) For uniaxial static compression creep performance, the cumulative creep deformation and residual permanent deformation of nano-TiO_2_/CaCO_3_ modified bituminous mixture exhibited lower values than the control base bituminous mixture. This indicated that bituminous mixture modified with nano-TiO_2_/CaCO_3_ had higher high-temperature rutting resistance. The reason is that the addition of nano-TiO_2_/CaCO_3_ can significantly improve the creep stiffness modulus and activation energy of bituminous mixture.
(3) The modified Burgers model can accurately characterize the cumulative strain of bituminous mixtures in the first two creep stages, as well as the influence of test temperature. The modified Burgers model can reflect the consolidation effect of bituminous mixture; that is, the creep growth rate of bituminous mixture gradually decreases in the actual creep process.
(4) According to the analysis of dynamic modulus and phase angle, the dynamic modulus of bituminous mixtures increased significantly as the frequency increased or the temperature decreased. Additionally, the phase angle of bituminous mixtures was more affected by the bituminous binder at lower temperatures or high-frequency loading. However, the mineral skeleton played an important role for the phase angle of bituminous mixture at high temperatures or low-frequency loading.
(5) The generalized Sigmoidal model can accurately grasp the characteristics of the relationship between dynamic modulus and reduced frequency. In addition, combined with the predicted dynamic modulus in a wider frequency range, the incorporation of nano-TiO_2_/CaCO_3_ can significantly enhance the high-temperature anti-rutting, and slightly improve the low-temperature anti-cracking of bituminous mixture.

## Figures and Tables

**Table 1:** Technical properties of 90# base bitumen.

Technical Properties		Methods	Values
Penetration	0.1 mm @ 25 °C	T0604	95.9
Ductility	cm @ 5 °C	T0605	12.8
	cm @ 10 °C		>100
Softening point	°C	T0606	43.0
Density	g/cm^3^ @ 15 °C	T0603	1.018
Dynamic viscosity	Pa∙s @ 60 °C	T0620	98.8
	Pa∙s @ 135 °C		0.294
RTFOT			
Mass loss	%	T0610	−0.189
Residual penetration ratio	% (@ 25 °C)	T0604	85.2

**Table 2:** Technical properties of nano-TiO_2_/CaCO_3_.

Technical Properties		Values
Appearance	—	White power
Bulk density	g/cm^3^	0.3
Average particle size	nm	300
Specific surface area	m^2^/g	10
Proportion	—	20% TiO_2_ + 80% CaCO_3_

**Table 3:** Technical properties of aggregates and mineral filler.

Technical Properties		Unit	Methods	Values
**Coarse Aggregate**				
Los Angeles abrasion value		%	T0317	16.8
Crushing value		%	T0316	13.4
Apparent specific gravity	13.2 mm	—	T0304	2.829
	9.5 mm			2.803
	4.75 mm			2.847
Water absorption	13.2 mm	%	T0304	0.65
	9.5 mm			0.36
	4.75 mm			0.88
Flat and elongated particle content		%	T0312	8.8
**Fine Aggregate**				
Apparent specific gravity		—	T0328	2.764
Sand equivalent		%	T0334	75
**Mineral Filler**				
Apparent density		t/m^3^	T0352	2.748
Hydrophilic coefficient		—	T0353	0.87
Water content		%	T0103	0.9
Plastic index		%	T0354	2
Granular composition	<0.6 mm	%	T0351	100
	<0.15 mm			98.6
	<0.075 mm			78.5

**Table 4:** Load levels at different temperatures.

**Temperature (°C** **)**	5	20	35	50
**Load range (kPa)**	700~1400	350~700	140~250	35~70

**Table 5:** Repeat loading cycles at different load frequencies.

**Load frequency (Hz** **)**	0.1	0.5	1	5	10	25
**Repeat loading cycle**	15	15	20	100	200	200

**Table 6:** The shift factor sat different temperatures.

Temperature (°C)	40	50	60	70	80
Base bitumen (control grup)	27.417	4.681	1	0.268	0.090
Nano-TiO_2_/CaCO_3_ bitumen	24.858	4.523	1	0.275	0.088

**Table 7:** The creep curve slope (*k*) and creep activation energy.

Bituminous Mixture Types	Creep Curve Slope (s^−1^)			Creep Activation Energy (J/mol)
	20 °C	35 °C	50 °C	
Base bitumen (control grup)	2.41 × 10^−5^	3.69 × 10^−5^	4.62 × 10^−5^	17,091.92
Nano-TiO_2_/CaCO_3_ bitumen	2.10 × 10^−5^	3.12 × 10^−5^	3.99 × 10^−5^	20,574.66

## Data Availability

The data presented in this study are available on request from the corresponding author.
